# MiR-181a-5p promotes osteogenesis by targeting BMP3

**DOI:** 10.18632/aging.204505

**Published:** 2023-02-03

**Authors:** Ze Long, Pengcheng Dou, Weiliang Cai, Minzhi Mao, Ren Wu

**Affiliations:** 1 Department of Orthopedics, The Second Xiangya Hospital of Central South University, Changsha, China

**Keywords:** miR-181a-5p, BMP3, MC3T3-E1, senile osteoporosis, osteogenesis

## Abstract

High-throughput microRNA (miRNA) sequencing of osteoporosis was analyzed from the Gene Expression Omnibus (GEO) database to investigate specific microRNAs that control osteogenesis. MiR-181a-5p was differentially expressed among healthy subjects and those with osteoporosis. Inhibitors and mimics were transfected into cells to modulate miR-181a-5p levels to examine the role in MC3T3-E1 functions. Alkaline phosphatase (ALP) staining and Alizarin Red S (ARS) staining were used for morphological detection, and proteins of ALP and Runt-related transcription factor 2 (RUNX2), as osteogenesis markers, were detected. During the osteogenic differentiation of MC3T3-E1, the transcription level of miR-181a-5p was significantly increased. The inhibition of miR-181a-5p suppressed MC3T3-E1 osteogenic differentiation, whereas its overexpression functioned oppositely. Consistently, the miR-181a-5p antagomir aggravated osteoporosis in old mice. Additionally, we predicted potential target genes via TargetScan and miRDB and identified bone morphogenetic protein 3 (BMP3) as the target gene. Moreover, the reduced expression of miR-181a-5p was validated in our hospitalized osteoporotic patients. These findings have substantial implications for the strategies targeting miR-181a-5p to prevent osteoporosis and potential related fractures.

## INTRODUCTION

Osteogenesis is a complex dynamic gene-modified program of osteoblasts that leads to the production of a collagenous mineralized matrix, playing a crucial role in bone homeostasis [1]. Bone formation controlled by osteoblast lineage cells involves multiple genetic and epigenetic regulation mechanisms, including transcriptional modification by M6A methylation and nucleosomes and chromatin architecture modification [2]. In addition to chromosome-related mechanisms, microRNAs (miRNAs) also control osteogenesis by targeting osteoblast-related mRNAs as post-transcriptional epigenetic regulation [3].

MiRNAs are single-stranded RNAs that have a length of 19–24 nucleotides [3, 4]. They are first transcribed from the genome into primary miRNAs before undergoing transformation into precursor and mature forms [4]. Numerous biological processes, such as apoptosis, the development of cancer, osteoblastogenesis and osteoclastogenesis, have been linked to microRNAs [5–7]. Previous studies have demonstrated the significance of miR-181a/b in the regulation of lung cancer angiogenesis, invasion, and metastasis [8, 9]. Additionally, via altering PBX1-mediated genes, miR-181a-5p modulates cellular ossification in the ligament [10]. In fibrous dysplasia, downregulation of miR-181a-5p impairs BMSC osteogenic differentiation [11]. Furthermore, miR-181a is regarded as one of the mitochondria-associated microRNAs during the osteogenic differentiation of human MSCs [12]. However, the connection between miR-181a-5p and osteogenesis is yet unidentified.

Thus, in this study, the expression of miR-181a-5p in subjects with osteoporosis was investigated after screening miRNAs with varying expression from a Gene Expression Omnibus (GEO) dataset. We tested whether it could control osteogenesis in both MC3T3-E1 cells and senile osteoporotic mice and further explored the potential mechanism.

## RESULTS

### GEO dataset analysis

**Figure 1:**
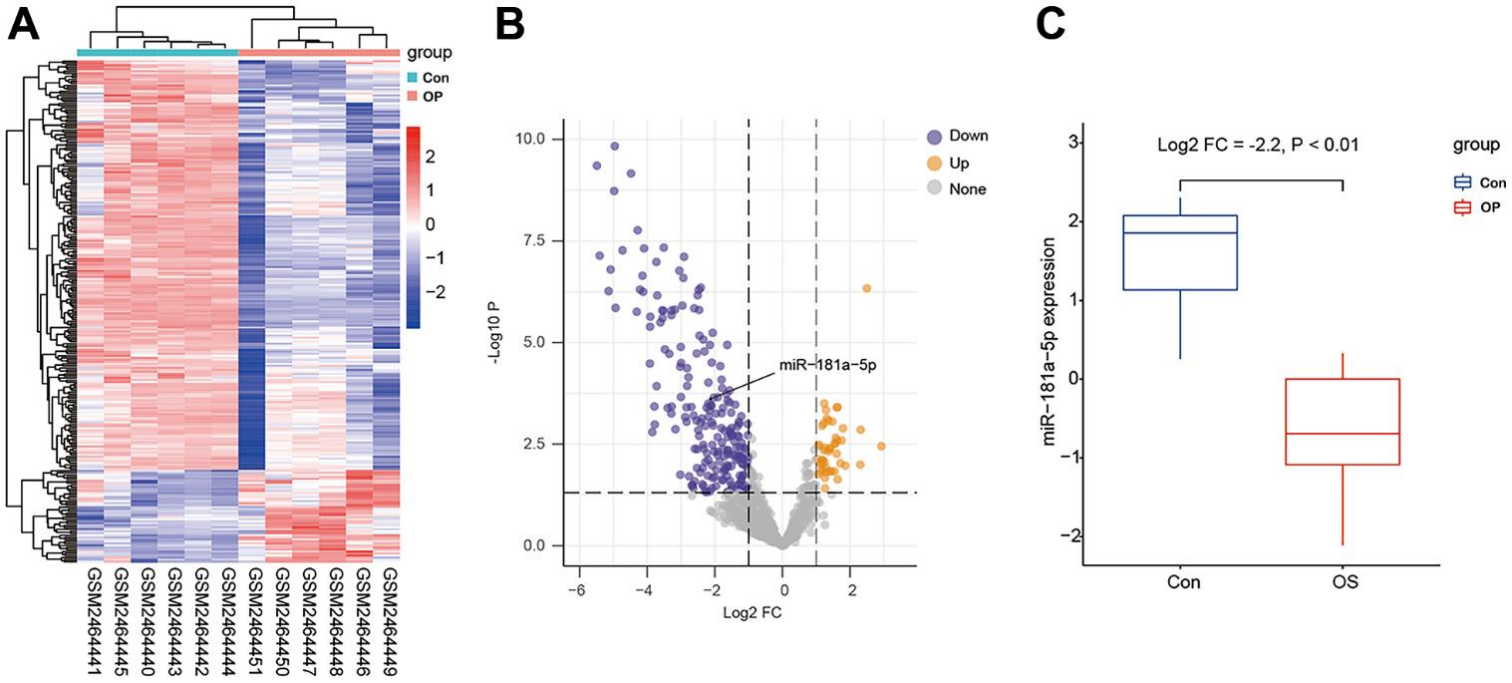
**Differentially expressed microRNAs in GSE93883.** (**A**) Heatmap of microRNA expression among six healthy (Con) and six osteoporotic (OP) samples. (**B**) A volcano plot of microRNAs by setting the cutoff as a log2 fold change of > 1 and a P value of < 0.5. Gray color indicates non-significant microRNAs, while yellow or purple color represents up- or down-regulated microRNAs. The black arrow indicates miR-181a-5p. (**C**) mRNA level of miR-181a-5p in healthy controls and osteoporotic patients in GSE93883.

**Figure 2:**
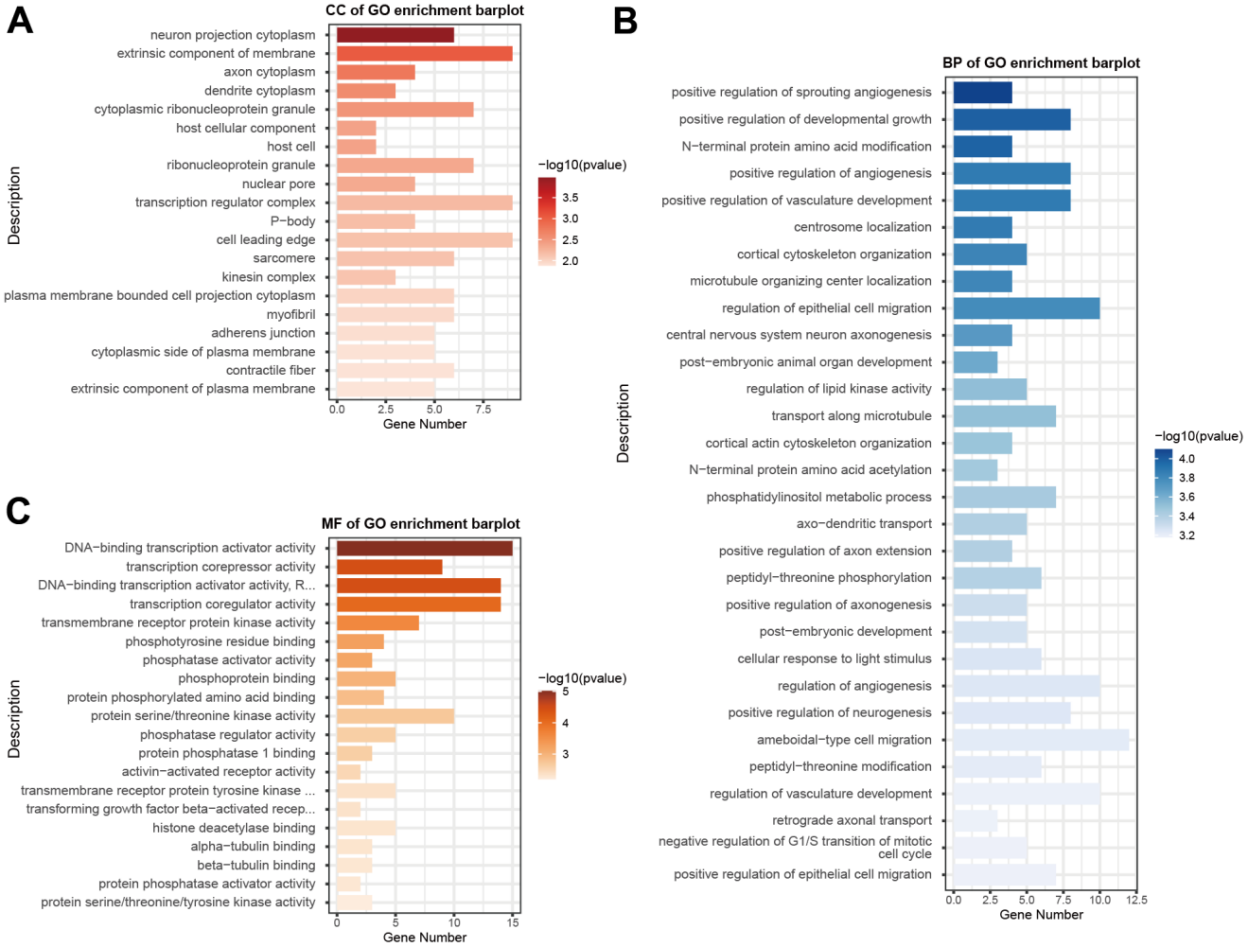
**GO functional enrichment analysis of differentially expressed microRNAs.** GO enrichment analysis was carried out using CC (**A**), BP (**B**), and MF (**C**). The x-axis shows enriched gene numbers and the color represents significance. GO terms are shown on the y-axis. GO, gene ontology; CC, cellular component; BP, biological process; MF, and molecular function.

The GSE93883 dataset was used for differentially expressed microRNA analysis based on microRNA arrays, including six healthy and six osteoporotic samples. By setting the cutoff as a log2 fold change of > 1 and a P value of < 0.5, a total of 123 microRNAs were differentially expressed (Figure 1A, 1B). Among these microRNAs, hsa-MiR-181a-5p was selected since its expression was significantly lower in patients with osteoporosis (Figure 1C). Gene ontology (GO) enrichment analysis was conducted, containing cellular component (CC), biological process (BP), and molecular function (MF) (Figure 2A–2C). MF analysis (Figure 2C) showed high enrichment of DNA-transcription activator and co-regulator activity, consistent with the recognition of a wide range of microRNA functions [6, 7]. BP analysis (Figure 2B) revealed that pathways such as development growth, regulation of angiogenesis, and cortical skeleton organization were highly enriched, indicating that these microRNAs were potentially involved in bone formation.

### Osteogenesis model in MC3T3-E1

We characterized the osteogenic differentiation model in MC3T3-E1 cells treated with an osteogenic induction culture medium for two weeks, as previously reported [13, 14]. The alkaline phosphatase (ALP) staining, which is a widely recognized marker for osteoblast activity [15], showed a significant ALP activity increase on day 14 compared to day 0 (Supplementary Figure 1A). For decades, calcium-rich deposits in cultured cells have been assessed using Alizarin Red S (ARS) staining [15]. Similarly, MC3T3-E1 cells on day 14 of differentiation demonstrated a remarkable signal of mineral deposition (Supplementary Figure 1B). In addition to cellular morphological change, Runt-related transcription factor 2 (RUNX2) is an essential controller for forming precursor osteoblasts and inducing osteogenesis, which is also considered a crucial differentiation marker protein [16]. The western blot was performed to detect ALP and RUNX2 protein levels, and results indicated a 2.1- and 2.5-fold increase in these protein levels, respectively, after osteogenic induction (Supplementary Figure 1C, 1D). The transcriptional level of miR-181a-5p rose at the end-point of MC3T3-E1 osteogenic differentiation (Supplementary Figure 1E). These data indicated that the *in vitro* model of osteogenic differentiation was induced, and the miR-181a-5p expression was remarkably altered during this period.

### Inhibition of miR-181a-5p suppressed osteogenic differentiation

**Figure 3:**
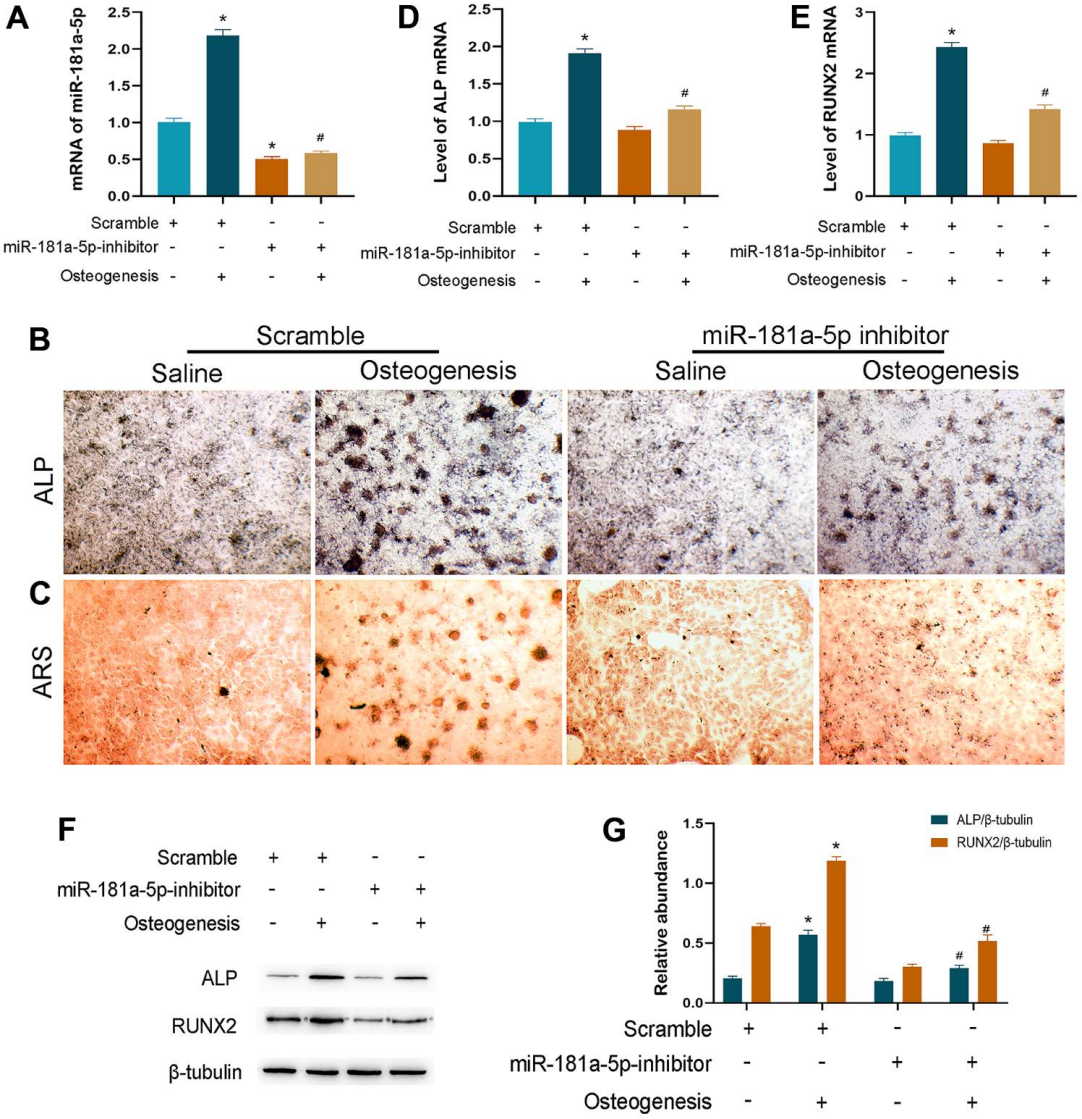
**Inhibition of miR-181a-5p suppressed osteogenic differentiation.** (**A**) miR-181a-5p expression in the scramble and inhibitor groups treated with saline or osteogenesis induction medium. (**B**, **C**) ALP and ARS staining of the scramble and inhibitor groups treated with saline or osteogenesis induction medium. (**D**, **E**) Transcriptional levels of ALP and RUNX2 by polymerase chain reaction (PCR). (**F**, **G**) ALP, RUNX2 and beta-tubulin are determined and quantified by densitometric evaluation of western blots, further normalized to beta-tubulin. All data represent mean ± s.e.m. (*n* = 6). *P<0.05 compared with the scramble group treated with saline. ^#^P<0.05 compared with the scramble group induced for differentiation.

Inhibitor transfection was performed to suppress the endogenous miR-181a-5p mRNA level while using scramble as the control. In the scramble group, the transcriptional level of the miRNA was increased during osteogenesis, which was in accordance with the induced primary osteogenic MC3T3-E1 cell model (Figure 3A). The suppression effect of inhibitor transfection was efficient and persistent throughout osteogenic differentiation, typically on day 14 (Figure 3A). These data verified the method proficiency of miR-181a-5p silencing.

Then, we observed the ALP activity and ARS-detected mineral deposition in two modified groups at the end-time point of osteogenesis. In comparison to the scramble group, miR-181a-5p inhibition significantly suppressed ALP and ARS staining signals (Figure 3B, 3C). Consistently, significant downregulations of ALP and RUNX2 were noticed when miR-181a-5p was inactivated (Figure 3D–3G). These results suggested that miR-181a-5p inhibition impaired MC3T3-E1 cell osteogenic differentiation and reduced extracellular mineral formation.

### Overexpression of miR-181a-5p enhanced osteogenic differentiation

**Figure 4:**
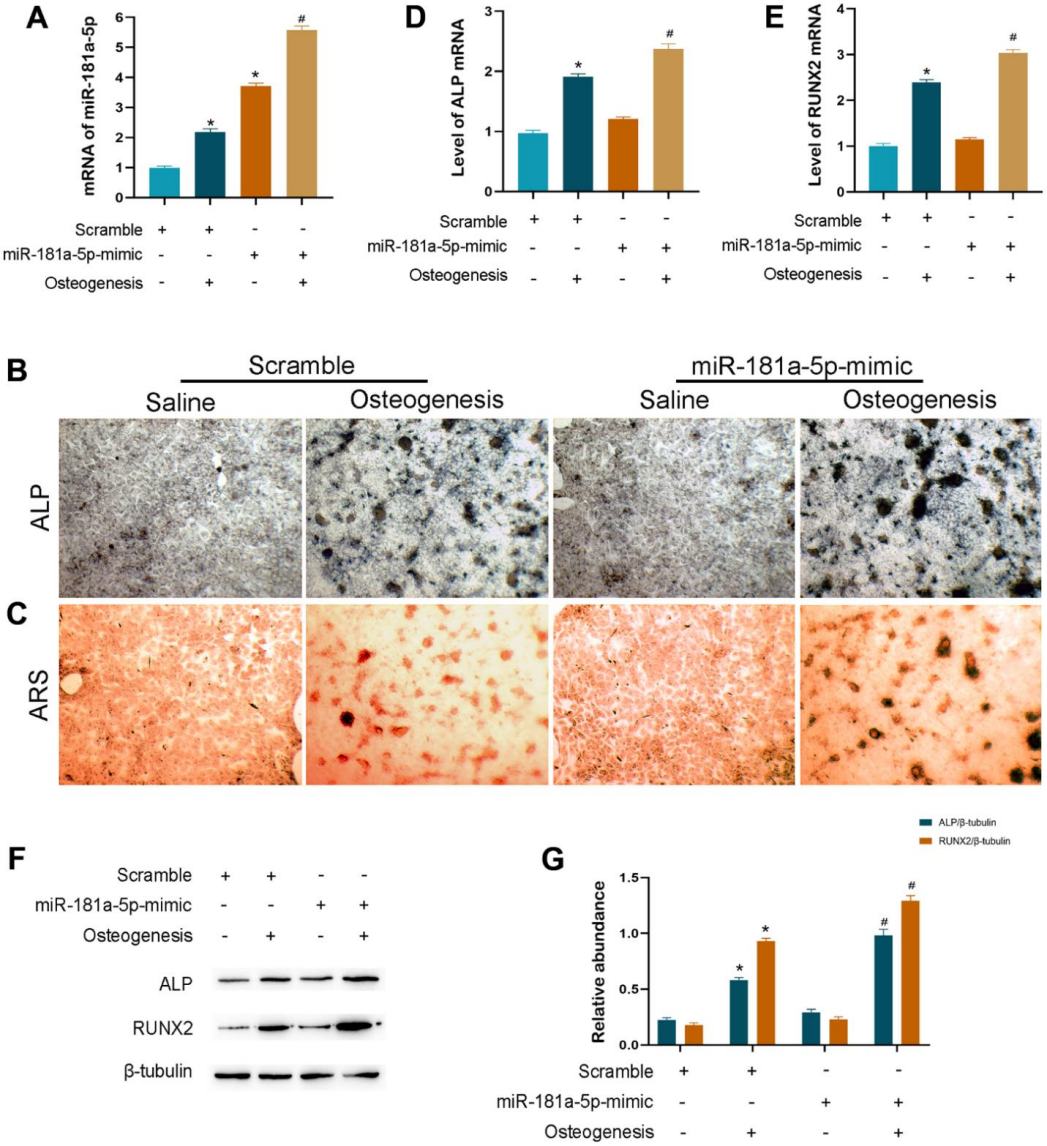
**Overexpression of miR-181a-5p enhanced osteogenic differentiation.** (**A**) miR-181a-5p expression in the scramble and mimic groups treated with saline or osteogenesis induction medium. (**B**, **C**) ALP and ARS staining of the scramble and mimic groups treated with saline or osteogenesis induction medium. (**D**, **E**) Transcriptional levels of ALP and RUNX2 by PCR. (**F**, **G**) ALP, RUNX2 and beta-tubulin are determined and quantified by densitometric evaluation of western blots, further normalized to beta-tubulin. All data represent mean ± s.e.m. (*n* = 6). *P<0.05 compared with the scramble group treated with saline. ^#^P<0.05 compared with the scramble group induced for differentiation.

We created the miRNA overexpressed cells by using mimic transfection as the contrary side of suppressed function to further validate the causal effect in osteogenic differentiation. The amount of miR-181a-5p mRNA was remarkably boosted in the mimic group, with a specifically stronger elevation after incubation (Figure 4A). Enrichment of miR-181a-5p greatly stimulated MC3T3-E1 cellular differentiation as assessed by ALP and ARS staining (Figure 4B, 4C). Additionally, ALP and RUNX2 were greatly upregulated in miR-181a-5p overexpressed cells (Figure 4D–4G). Combined with the inhibitor effect, all of these findings revealed that it positively regulated osteoblast cellular osteogenesis.

### Inhibition of miR-181a-5p aggravated senile osteoporosis in mice

**Figure 5:**
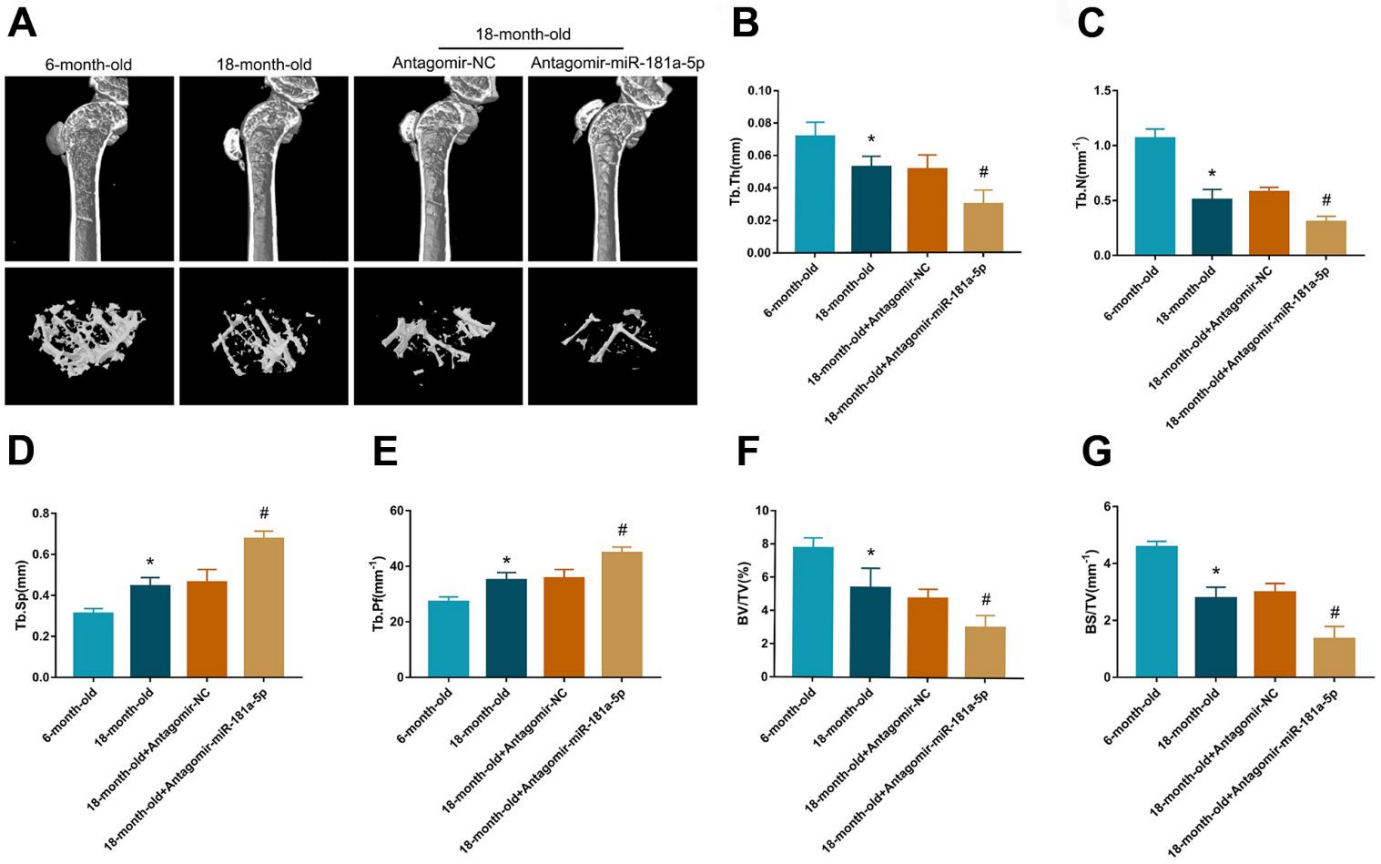
**Inhibition of miR-181a-5p aggravated senile osteoporosis in mice.** (**A**) Representative 3D reconstruction images of microarchitecture in the mice tibia. Groups are divided as follows: 6-month-old mice, 18-month-old mice, 18-month-old mice treated with antagomir-negative control (NC) and 18-month-old mice treated with antagomir-miR-181a-5p. (**B**–**G**) Micro-CT analysis includes trabecular thickness (Tb.Th), trabecular number (Tb.N), trabecular separation (Tb.Sp), trabecular pattern factor (Tb.Pf), bone volume fraction (BV/TV) and bone surface density (BS/TV). All data represent mean ± s.e.m. (n = 5). *P<0.05 compared with the 6-month-old mice group. ^#^P<0.05 compared with the group of 18-month-old mice treated with antagomir-NC.

The senile osteoporosis mouse model is a popular method for studying age-related declines in osteogenic potential [17, 18]. We used 6- and 18-month-old mice to establish the senile osteoporosis model with tibia trabecular bone loss, which was mainly characterized by microcomputed tomography (CT). Application of the antagomir-miR-181a-5p and the antagomir-control were conducted for four weeks to further validate our findings *in vivo*. Age-related trabecular bone loss and microarchitecture deterioration were presented in mice aged 18 months compared to those aged six months (Figure 5A). Suppression of miR-181a-5p further decreased trabecular bone mass and worsened trabecular microarchitecture, with declines in trabecular thickness, number, bone volume fraction, and bone surface density, as well as enhancements in trabecular separation and pattern factor (Figure 5A–5G). These data implied that miR-181a-5p blockade aggravated senile osteoporosis in mice.

### MiR-181a-5p targeted on BMP3

**Figure 6:**
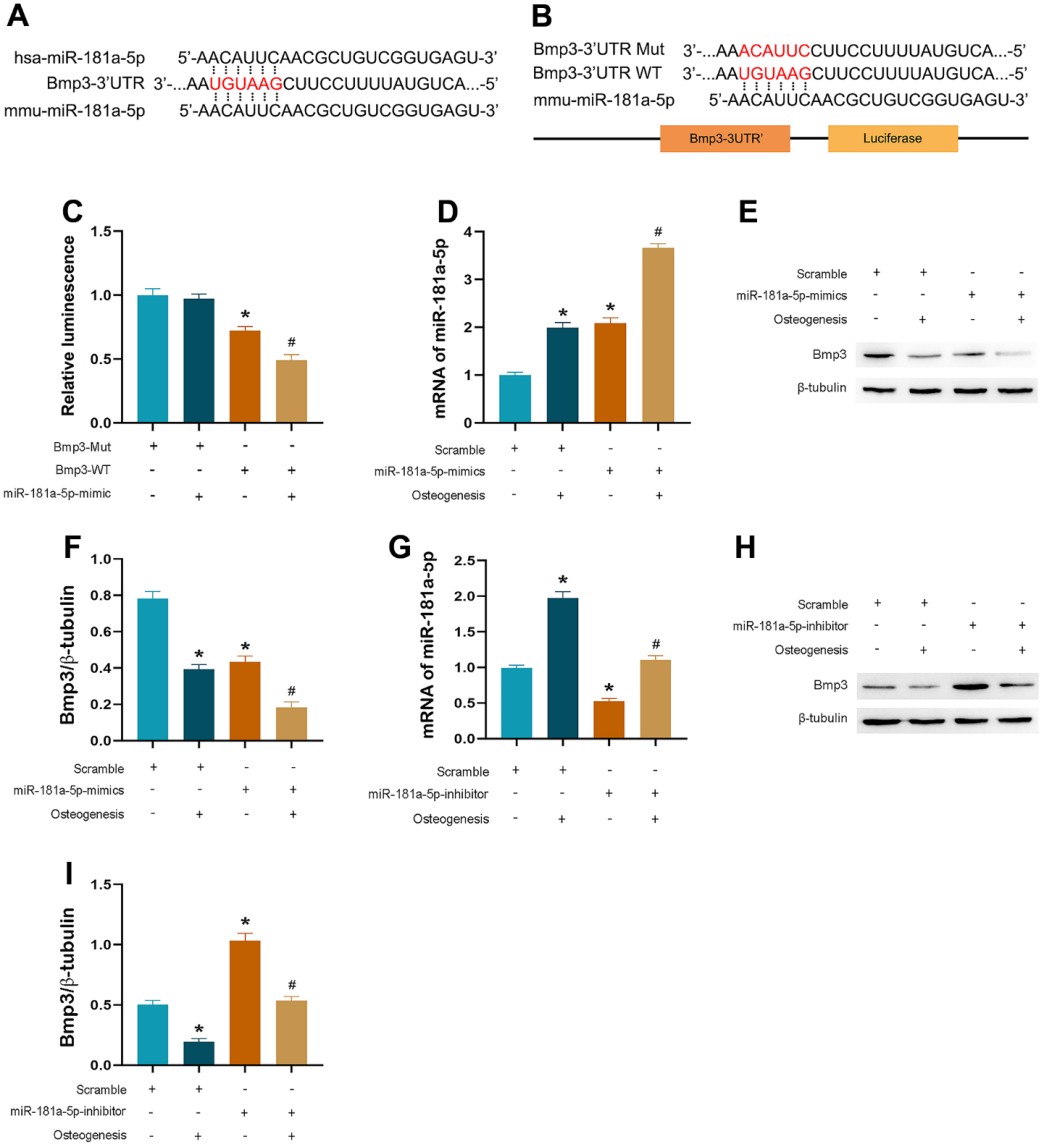
**miR-181a-5p targeted on BMP3.** (**A**, **B**) The sequence of BMP3-3’ UTR WT or BMP3-3’ UTR Mut luciferase constructs. (**C**) Relative luminescence of scramble and miR-181a-5p mimic transfected cells which are contransfected with BMP3-3’ UTR WT or BMP3-3’ UTR Mut luciferase constructs. Data represent mean ± s.e.m. of the ratio to the value of scramble in BMP3-Mut transfected cells. *P<0.05 compared with the scramble and BMP3-Mut contransfected cells. # P<0.05 compared with the scramble and BMP3-WT contransfected cells. (**D**) PCR verification of miR-181a-5p mimic transfection efficiency. (**E**, **F**) BMP3 and beta-tubulin protein levels in miR-181a-5p mimic transfected cells. BMP3 is normalized to beta-tubulin. *P<0.05 compared with the scramble group treated with saline. #P<0.05 compared with the miR-181a-5p mimic group treated with saline. (**G**) PCR verification of miR-181a-5p inhibitor transfection efficiency. (**H**, **I**) BMP3 and beta-tubulin protein levels in miR-181a-5p inhibitor transfected cells. BMP3 is normalized to beta-tubulin. *P<0.05 compared with the scramble group treated with saline. #P<0.05 compared with the miR-181a-5p inhibitor group treated with saline. All data represent mean ± s.e.m. (n = 6).

By utilizing online databases (TargetScan and miRDB), we identified target genes of miR-181a-5p. Among all the candidates, we further explored bone morphogenetic protein 3 (BMP3), which is the most abundant BMP and has been reported as an important negative regulator of osteoblastogenesis and bone mass [19]. Based on the luciferase assay, miR-181a-5p interacted with the complementary site within the 3’UTR of BMP3 (Figure 6A–6C). The effectiveness of mimic transfection was verified (Figure 6D). Results showed that miR-181a-5p targeted BMP3 in MC3T3-E1 cells, given the significant downregulation of BMP3 at the protein level in overexpressed cells compared to the scramble group, especially after induction (Figure 6E, 6F). Similarly, the inhibitor group represented an increased BMP3 protein level (Figure 6G–6I). These discoveries revealed that the potential gene, *BMP3*, was verified as the target.

### Validation of miR-181a-5p levels in osteoporotic patients

**Figure 7:**
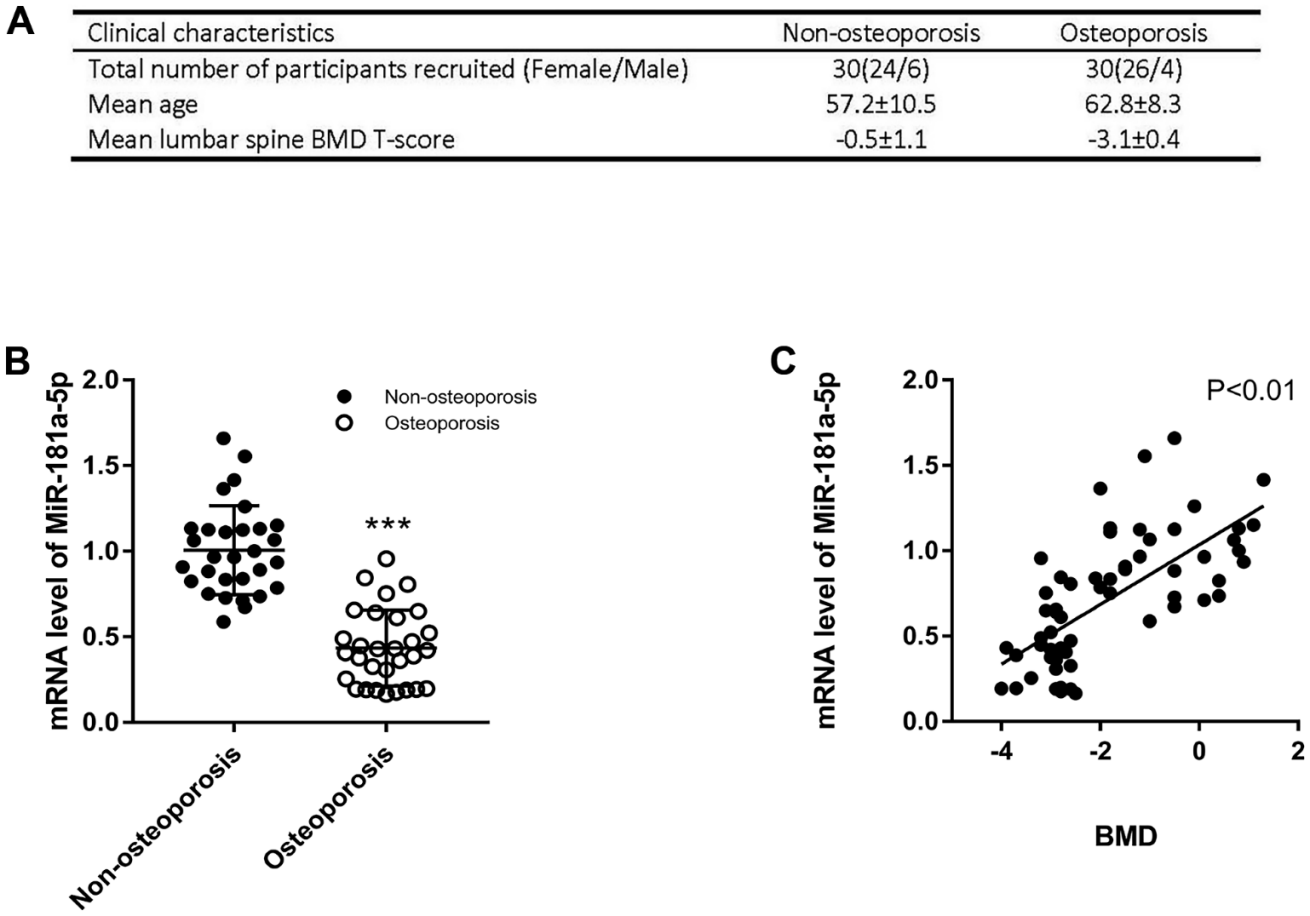
**Validation of miR-181a-5p level in osteoporotic patients.** (**A**) The clinical information of recruited healthy volunteers and patients includes the number of male/female, the mean age and the mean lumbar spine BMD T-score. (**B**) mRNA levels of miR-181a-5p in healthy volunteers and osteoporosis patients. Data represent mean ± s.e.m. (*n* = 30). ***P<0.001. (**C**) Linear regression of BMD and miR-181a-5p mRNA levels in the whole cohort (*n* = 60).

Individuals were recruited in our hospital following the widely recognized definition of osteoporosis [20]. Based on our clinical cohort of 30 osteoporotic and 30 non-osteoporotic patients, we extracted RNA from plasma following a permitted protocol and detected miR-181a-5p. Related information and characteristics were presented in Figure 7A. In accordance with the results of the GEO dataset, it was remarkably reduced in osteoporotic patients, as assessed by polymerase chain reaction (PCR) (Figure 7B). Moreover, a substantial positive correlation between the expression and the spine bone mineral density (BMD) was found in Figure 7C. Taken all these together, miR-181a-5p decreased in osteoporotic patients and was positively correlated with BMD.

## DISCUSSION

Osteoporosis is a global health and economic burden [21]. Over 3 million fractures are anticipated to result from osteoporosis in the US by 2025, costing $25.3 billion annually [22, 23]. Osteogenic differentiation and mineralization are regarded as contributors to normal bone homeostasis, the dysfunction of which results in osteoporosis and an increased risk of fractures [24]. In the current study, we selected miR-181a-5p, which had a different expression in subjects with osteoporosis from a GEO dataset, and investigated the function of this miRNA *in vitro* and *in vivo*. The data in this study implied that it promoted cellular osteogenic differentiation and matrix calcium mineralization, and the antagomir aggravated senile osteoporosis in mice. Potential target genes were scrutinized, and BMP3 was identified as the target. Furthermore, we validated the downregulation of this miRNA in our osteoporosis cohort and found that it was positively correlated with BMD. Thus, miR-181a-5p was found to positively regulate osteogenesis via BMP3 and was lowly expressed in osteoporotic patients.

MC3T3-E1 osteoblasts are utilized to study the mechanism of osteoporosis, given their routine application of studying osteogenic differentiation and mineralization *in vitro* [13, 14]. Biomarkers, including ALP and RUNX2, are tested during this process. ALP is one of the most reliable markers for osteogenic differentiation and is expressed early in bone cells [15]. Bone mineralization requires ALP activation [25]. RUNX2, a member of the RUNT transcription factor family, functions as a master regulator of osteogenesis [26]. In detail, bone formation is severely impaired in RUNX2-deficient mice [27, 28]. The modulation of RUNX2's transcriptional activity by YAP1 and SMAD also contributes to osteogenesis [29, 30]. Animal models of senile osteoporosis have been used to advance knowledge of cellular mechanisms in osteogenesis [17]. A significant amount of bone is lost due to decreased osteoblast differentiation ability during the process of aging [18].

Multiple members of the miR-181 family have been implicated in various disorders [9, 31]. Regarding the topic of this study, some of them have been demonstrated to have critical roles in bone growth as well as chondrocyte function [32–35]. Enrichment of miR-181a, for example, boosts osteogenesis *in vitro* via PTEN/PI3K/AKT pathway or TGF-β signaling [32, 33]. Additionally, miR-181a suppresses cartilage cellular growth by altering CCN1 and ACAN [34]. MiR-181b has been illustrated to restrain cartilage development *in vivo* and *in vitro* [35]. Consistent with these data, we found that miR-181a-5p functioned as a causal regulator of osteogenesis in both MC3T3-E1 cells and old mice.

BMP3 serves as a negative controller of osteoblast differentiation and bone density [19]. It restrains osteoblast differentiation of bone marrow stromal cells by interacting with Acvr2b [36]. Furthermore, BMP3 serves as a target of miR-450b to regulate bone formation [37]. A recent study has presented that bone mineralization was enhanced in BMP3−/− mice, suggesting that BMP3 could influence long bone development in mice [38]. In this study, experiments of the luciferase assay showed that the suppression of miR-181a-5p increased luciferase activity while the overexpression decreased it. Additionally, the role of BMP3 as the target gene was verified by western blot.

In recent years, microRNAs have been considered as promising therapeutics for osteoporosis [39]. Clinical studies are already being conducted for novel pharmaceuticals that target miRNAs to cure disease [40]. This study may provide a rationale for a strategy targeting miR-181a-5p to prevent osteoporosis and potential related fractures.

## MATERIALS AND METHODS

### Bioinformatic analysis

The GSE93883 dataset was accessible at the Gene Expression Omnibus (GEO) database (https://www.ncbi.nlm.nih.gov/geo/). Using TargetScan (https://www.targetscan.org/) and miRDB (http://mirdb.org/), target mRNAs for microRNAs were scrutinized. The R program (version 3.5.2, Austria) was performed with packages including limma, factoextra, pheatmap, EnhancedVolcano, and ggpubr.

### Cell culture and treatment

MC3T3-E1 cells were sourced from the Chinese Academy of Sciences’ cell library (China, originally from ATCC, USA)). Osteogenic differentiation was induced by culturing MC3T3-E1 cells with induction medium for 14 days as previously described [13, 14]. The inhibitor and mimic were obtained from RiboBio Co. (Guangzhou, China), and transfection experiments were conducted utilizing the Lipofectamine 2000 reagent (Thermofisher, USA) as instructed by the manufacturer.

### Alkaline phosphatase (ALP) and Alizarin Red S (ARS) staining

ALP activity analysis was carried out utilizing Alkaline Phosphatase Staining Kit (Abcam, ab284936). As directed by the manufacturer, cells were fixed in 95% methanol for 10 minutes, stained with ALP staining reagent for 15 minutes, and then rinsed with wash buffer. Before being treated for 5 minutes with Alizarin Red Solution 2% (Solarbio, G1450), cells were fixed in 95% methanol for 10 minutes. Then cells were rinsed and imaged under a microscope to estimate the extracellular matrix calcification.

### Real-time quantitative PCR

Total RNA extraction was carried out with Trizol (AG, 21101) following the manufacturer’s protocols. The Reverse Transcriptase M-MLV (AG, 11705) was then used to convert the extracted total RNA into cDNA. Primer sequences of *RUNX2* were: forward: 5′ CTCACTACCACACCTACCTG 3′ and reverse: 5′ TCAATATGGTCGCCAAACAGATTC 3′. Primer sequences of *ALP* were: forward: 5′ CCACGTCTTCACATTTGGTG 3′ and reverse: 5′-AGACTGCGCCTGGTAGTTGT-3′. The miRNA first-strand cDNA synthesis kit (Accurate Biotechnology, Hunan) was performed to reverse-transcribe miRNA. The miR-181a-5p-specific forward primer was AACATTCAACGCTGTCGGTGAGT, and U6 served as a standardization control. Quantitative reverse transcriptase PCR was carried out, and the relative standard curve method was performed to assess the results of the PCR data analysis.

### Western blot analysis

Western blot performance was assessed as previously published [41]. Antibodies including Anti-RUNX2 (Abcam, ab236639), Anti-ALP (CST, 8681), Anti-BMP3 (Bioworld Technology, BS5629), and Beta-Tubulin (Affinity, T0023) were applied to determine RUNX2, ALP, BMP3 and beta-Tubulin, respectively.

### Mice and treatment

The C57BL/6 wild type mice aged 6 months and 18 months were obtained from Cavens Co. (Changzhou, China). The 18-month-old mice received antagomir-miR-181a-5p or antagomir-negative control (10 mg/kg body weight) with the osteoblast-targeted delivery system by tail vein injection twice per week for four weeks [42].

### Micro-CT analysis

Tibias were scanned through the micro-CT system (Skyscan, Bruker). The scanning resolution was set at 18 μm and 3D reconstruction of trabecular bone was used by Ctvol software. Parameters computed from these data include trabecular thickness (Tb.Th), trabecular number (Tb.N), trabecular separation (Tb.Sp), trabecular pattern factor (Tb.Pf), bone volume fraction (BV/TV) and bone surface density (BS/TV) [38, 42].

### Reporter gene assay

The BMP3 wild type and 3′-UTR mutation which included the binding site of miR-181a-5p were developed, and all transfections were conducted in 293T cells. As directed by the instruction from Luciferase Assay System (Promega, USA), cells were lysed and assayed for the luciferase activity following incubation for 48 hours.

### Clinical samples

Patients over the age of 18 who were capable of giving consent on their own, were included into the osteoporosis cohort while healthy volunteers as the control group. Patients under the age of 18, incapable of giving consent on their own, or afflicted with a condition unrelated to osteoporosis were excluded. The written consents were obtained from all the participants. RNA extraction from plasma samples was carried out with Trizol, then restored at -80° C.

### Statistical analysis

All data were presented as the means ± s.e.m. The student's t-test was utilized to evaluate the statistical significance between groups. P<0.05 was considered to be statistically significant.

## Supplementary Material

Supplementary Figure 1
