# Supplementary material for: MiR-181a-5p promotes osteogenesis by targeting BMP3
Source: Aging (Albany NY). 2023 Feb 3;15(3):734–47. doi: 10.18632/aging.204505 (PMC9970307; doi:10.18632/aging.204505)
Supplement: Supplementary Figure 1 [file aging-15-204505-s001.pdf]

SUPPLEMENTARY FIGURE

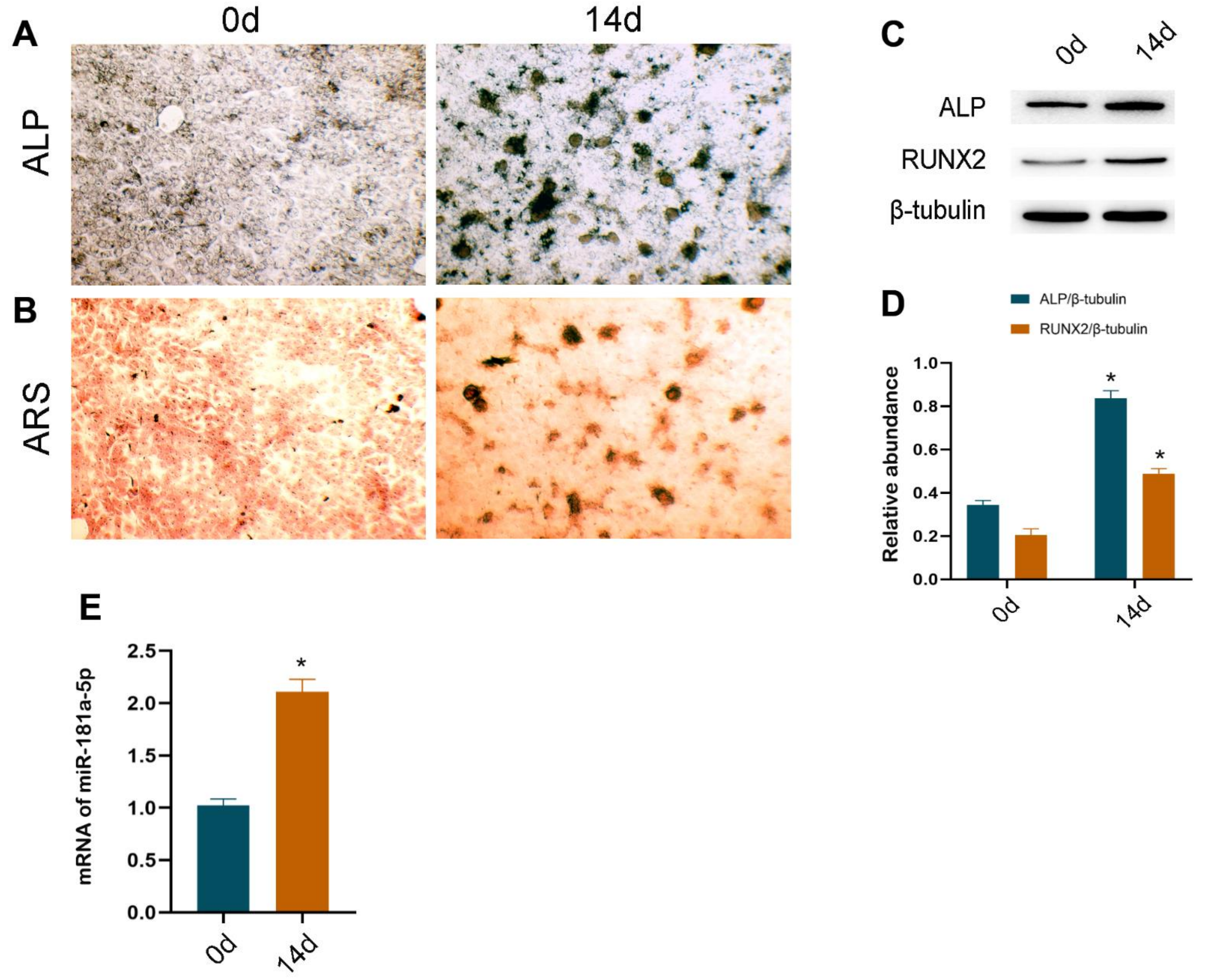

Supplementary Figure 1. Osteogenesis induced model in MC3T3-E1 cells. (**A**, **B**) ALP and ARS staining of MC3T3-E1 wild type cells at the start point (0 day) and end point (14 days). (**C**, **D**) ALP, RUNX2 and beta-tubulin are determined and quantified by densitometric evaluation of western blots, further normalized to beta-tubulin. (**E**) expression of miR-181a-5p in MC3T3-E1 cells induced for osteogenic differentiation. All data represent mean ± s.e.m. (n = 6). *P<0.05 compared to day 0.
